# Susceptibility of Bifidobacteria of Animal Origin to Selected Antimicrobial Agents

**DOI:** 10.1155/2011/989520

**Published:** 2011-04-05

**Authors:** Sigrid Mayrhofer, Christiane Mair, Wolfgang Kneifel, Konrad J. Domig

**Affiliations:** Department of Food Sciences and Technology, Institute of Food Sciences, BOKU-University of Natural Resources and Life Sciences, Muthgasse 18, 1190 Vienna, Austria

## Abstract

Strains of the genus *Bifidobacterium* are frequently used as probiotics, for which the absence of acquired antimicrobial resistance has become an important safety criterion. This clarifies the need for antibiotic susceptibility data for bifidobacteria. Based on a recently published standard for antimicrobial susceptibility testing of bifidobacteria with broth microdilution method, the range of susceptibility to selected antibiotics in 117 animal bifidobacterial strains was examined. Narrow unimodal MIC distributions either situated at the low-end (chloramphenicol, linezolid, and quinupristin/dalfopristin) or high-end (kanamycin, neomycin) concentration range could be detected. In contrast, the MIC distribution of trimethoprim was multimodal. Data derived from this study can be used as a basis for reviewing or verifying present microbiological breakpoints suggested by regulatory agencies to assess the safety of these micro-organisms intended for the use in probiotics.

## 1. Introduction

Probiotics are generally defined as “live micro-organisms which confer a health benefit on the host when administered in adequate amounts” [1]. There is considerable interest in probiotics for a variety of medical conditions, and millions of people around the world consume probiotic medications or foods daily for perceived health benefits [2]. Next to lactobacilli, members of the genus *Bifidobacterium* (*B.*) are frequently incorporated in probiotic products [3]. Although they have generally been regarded as safe (GRAS), there are theoretical concerns regarding their safety [2]. These concerns include the potential for transmigration and consequently the occurrence of diseases [2]. Hence, bifidobacteria have already been isolated from various clinical samples and reported as potential pathogens [4, 5]. Additionally, the potential transfer of antibiotic resistance genes from probiotic bacteria to commensals or potential pathogens within the gastrointestinal flora is taken very seriously [6, 7].

Thus, microorganisms used as probiotics for humans or additives in animal nutrition should not contain transferable antimicrobial resistance determinants [8–10]. Hence, the Panel on Additives and Products or Substances Used in Animal Feed (FEEDAP) of the European Food Safety Authority (EFSA) has defined criteria for the assessment of antimicrobial resistance of bacterial strains used as feed additives [11]. According to this panel, all bacteria intended for use as feed additives in Europe must be examined to ensure the susceptibility of the component strains to a relevant range of antibiotics [11]. Additionally, EFSA has proposed the use of the Qualified Presumption of Safety (QPS) status as a safety assessment tool for microorganisms added to food and feed. Within the QPS approach, which is a system similar in concept and purpose to the GRAS definition used in the USA, but modified to take account of different regulatory practices in Europe, the absence of acquired antibiotic resistance traits has to be confirmed for all strains of species with QPS status [12, 13]. 

In contrast to bacteria with clinical significance, standard procedures and breakpoints have been poorly validated for antimicrobial susceptibility testing of non pathogenic bacteria [14]. Because of missing standardized protocols and susceptibility data for lactic acid bacteria and bifidobacteria, risk assessment of these industrially important bacteria has been complicated in the past. The development of the lactic acid bacteria susceptibility test medium [15] proved to be a first major step forward to establish a standardized method for lactic acid bacteria and bifidobacteria. Meanwhile, this medium has been frequently applied for antimicrobial susceptibility testing of bifidobacteria [16–20]. Based on the use of this medium, standard operating procedures for antimicrobial susceptibility testing of bifidobacteria have been proposed [21] and were recently made available as ISO 10932/IDF 233 standard [22]. 

The occurrence of antimicrobial resistance in animal strains of *B. animalis*, *B. pseudolongum,* and *B. thermophilum* to seven antibiotics (i.e. ampicillin, clindamycin, erythromycin, gentamicin, streptomycin, tetracycline, and vancomycin) has been already determined [16, 17]. As up to 13 antibiotics were proposed by EFSA at the time of investigation [23], the susceptibility of strains of these *Bifidobacterium* species to the remaining antimicrobial agents chloramphenicol, kanamycin, linezolid, neomycin, quinupristin/dalfopristin, and trimethoprim was examined within this study. The obtained data may serve as basis for the definition of microbiological breakpoints for bifidobacteria. Furthermore, data could be used to eradicate of bifidobacteria from infections, although little is known about their pathogenic potential [4]. Comparing antimicrobial susceptibility data of human and animal strains, which were tested with the same medium, resistances were more prevalent in strains of animal origin [24]. The development of bacterial resistance in livestock may be favored due to the use of antibiotics throughout whole periods of life and widely used prophylaxis with medicated feeding stuff at low doses [25]. Thus, the spread of antimicrobial resistances in bifidobacteria could be better predicted by investigating animal strains.

## 2. Materials and Methods

### 2.1. Bacterial Strains and Growth Conditions

A total of 112 bifidobacteria of animal origin, isolated during the EU project BIFID (CT-2000-00805) [26], were included in this study belonging to the species: *B. animalis* (*n* = 8), *B. pseudolongum* (*n* = 33), and *B. thermophilum* (*n* = 71). The identification of the isolates at strain and species level was previously described by Mättö et al. [16] and Mayrhofer et al. [17]. The following BCCM/LMG microbial collection strains (University Gent, Belgium) were additionally tested as reference microorganisms: *B. pseudolongum* subsp. *globosum* LMG 11569 (ATCC 25865), *B. pseudolongum* subsp. *globosum* LMG 11614 (ATCC 25864), *B. pseudolongum* subsp. *pseudolongum *LMG 11594, *B. thermophilum* LMG 21813 (ATCC 25525; type strain), and *B. thermophilum *LMG 11574 (ATCC 25866).

Bacteria were maintained at −80°C, resuscitated in MRS broth (Oxoid, Hampshire, UK) supplemented with 0.5 g/liter cysteine-HCl (AppliChem, Darmstadt, Germany), and sub-cultured on the Lactic acid bacteria Susceptibility test Medium for bifidobacteria (LSM-C). This medium consists of 90% Isosensitest broth (Oxoid), 10% MRS broth (Oxoid), and 1.5% Agar Bacteriological (Oxoid) supplemented with 0.3 g/liter cysteine-HCl (LSM-C) [15]. All incubations were performed in an anaerobic cabinet (80% N_2_, 10% CO_2_ and 10% H_2_; Scholzen Technik, Switzerland) at 37°C.

### 2.2. Antimicrobial Susceptibility Testing

The minimum inhibitory concentrations (MICs) of the antimicrobial agents chloramphenicol, kanamycin, linezolid, neomycin, quinupristin/dalfopristin, and trimethoprim were determined by broth microdilution according to the ISO 10932/IDF 233 standard [22] with minor modifications. With the exception of quinupristin/dalfopristin (Sanalog, Kist, Germany) and linezolid (Pfizer, New York, USA), all antibiotics originated from Sigma-Aldrich (Daint Louis, Missouri, USA). All antibiotics except for chloramphenicol and trimethoprim were dissolved in water for preparing stock solutions of 1280 *μ*g/mL. To dissolve chloramphenicol 95% ethanol was needed, whereas 0.05 M HCl was required for trimethoprim. These solvents were used in volumes as low as possible, and water was finally added to receive the desired volume of the stock solution. Subsequently, stock solutions were diluted in LSM-C broth to obtain solutions with preliminary concentrations in the range of 0.25–256 *μ*g/mL. Of these, 50 *μ*l were dispensed in the wells of the microtiter plates. 

Bacterial inocula were prepared by suspending colonies from 48 h incubated LSM-C medium to 5 mL 0.85% NaCl solution. Subsequently, inocula were adjusted to McFarland standard 1 and diluted 1 : 500 in LSM-C broth for inoculation of microdilution plates by adding 50 *μ*l of diluted inoculum to each well. This resulted in a final antibiotic concentration of 0.12–128 *μ*g/mL. 

After incubating plates under anaerobic conditions at 37°C for 48 hours, the MIC value was read as the lowest concentration of an antimicrobial agent in which visible growth was inhibited.

The accuracy of susceptibility testing was monitored by parallel use of the quality control strain *Enterococcus faecalis* ATCC 29212.

## 3. Results and Discussion

**Figure 1:**
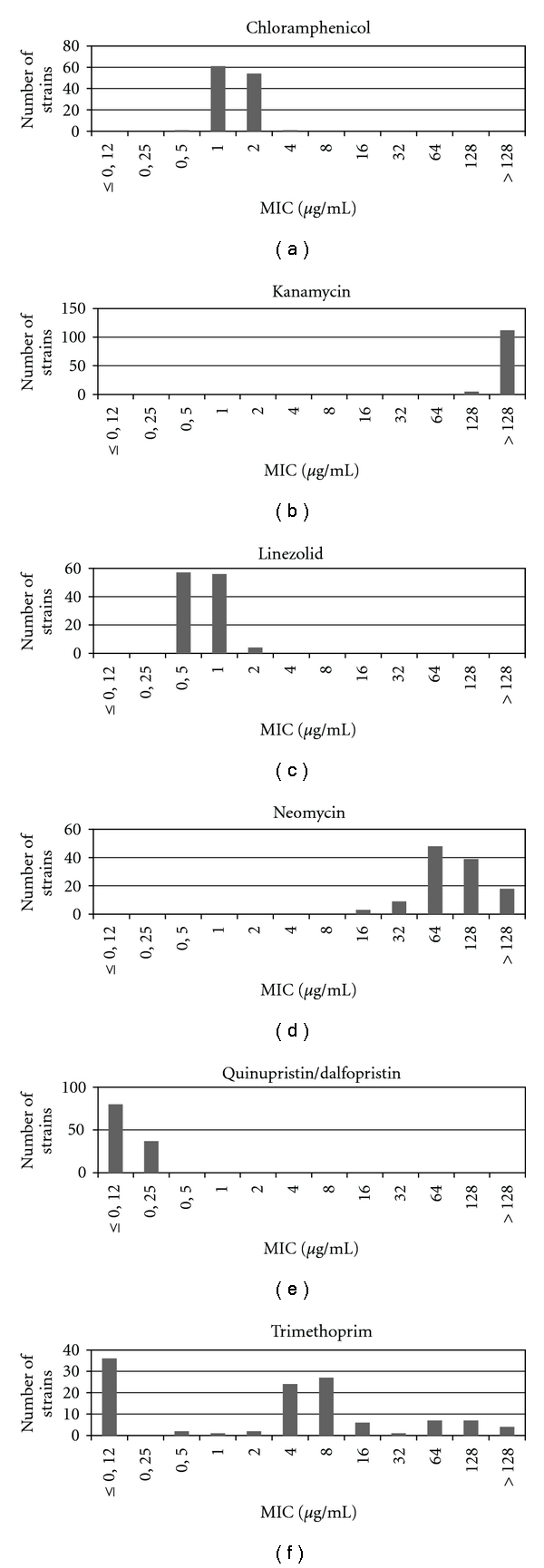
Distribution of minimum inhibitory concentrations (MICs) for (a) chloramphenicol, (b) kanamycin, (c) linezolid, (d) neomycin, (e) quinupristin/dalfopristin, and (f) trimethorpim in 8 *B. animalis*, 36. *B. pseudolongum,* and 73 *B. thermophilum* strains as determined with the microdilution broth method using LSM-C medium.

The antimicrobial susceptibility of 8 *B. animalis*, 36 *B. pseudolongum,* and 73 *B. thermophilum* strains, including five reference strains, to chloramphenicol, kanamycin, linezolid, neomycin, quinupristin/dalfopristin, and trimethoprim is summarized in Table 1. MICs (*μ*g/mL) are reported in terms of the MIC range, MIC_50_ (MIC that inhibited 50% of the tested strains), and MIC_90_ (MIC that inhibited 90% of the tested strains). Accordingly, no marked difference in the MIC distributions between the different species was observed for chloramphenicol, kanamycin, linezolid, neomycin, and quinupristin/dalfopristin. For these antimicrobial agents narrow unimodal MIC distributions either at the low-end concentration range (i.e., for chloramphenicol, linezolid, and quinupristin/dalfopristin) or at the high-end concentration range (i.e., for kanamycin and neomycin) were determined (Figures 1(a)–1(e)). A unimodal distribution describes a population, which is either uniformly susceptible (i.e., for chloramphenicol, linezolid, and quinupristin/dalfopristin) or resistant (i.e., for kanamycin and neomycin) [27, 28]. Next to a unimodal distribution MIC values, obtained by susceptibility testing of a defined population of strains, can also follow a bimodal or multimodal distribution [29]. For trimethoprim a multimodal distribution with three different subpopulations was identified: one with very low MICs (≤0.12 *μ*g/mL), another one with higher MICs (0.5–16 *μ*g/mL), and a last one with high MICs (32–>128 *μ*g/mL) (Figure 1(f)). While *B. animalis* strains generally had lower trimethoprim MICs (≤0.12 *μ*g/mL), the highest MICs were detected for *B. thermophilum* strains (0.5–>128 *μ*g/mL). The MIC values of *B. pseudolongum* strains were between ≤0.12 *μ*g/mL and 32 *μ*g/mL.

In order to allow the interpretation of antimicrobial susceptibility profiles of bifidobacteria used in food and feed applications, microbiological breakpoints are needed for categorizing susceptible or resistant strains. Especially bimodal distributions of MIC values play an important role in determining the microbiological breakpoint. Nevertheless, also unimodal and multimodal distributions can support the definition of microbiological breakpoints to distinguish strains with acquired resistance from the native, susceptible population. Beside this, MICs for determining microbiological breakpoints are only meaningful when the methods and conditions of the test are known [30]. It is for this reason that the development of an international standard reference method for determining MICs for bifidobacteria was recently proposed [22]. Thus, data obtained in this study, following the newest method developments concerning antimicrobial susceptibility testing of bifidobacteria, can be used as a basis for reviewing or verifying present microbiological breakpoints for bifidobacteria to assess the safety of microorganisms intended for use in food and feed applications.

According to the literature, bifidobacteria are usually sensitive to chloramphenicol. Normal MICs to this antimicrobial agent using various test techniques and media have been reported to range between 0.5 and 8 *μ*g/mL [31–37]. Only once in the literature, five strains belonging to the species *B. infantis*, *B. longum,* and *B. suis* as well as one *Bifidobacterium* sp. isolate with higher MICs up to 64 *μ*g/mL were detected by Kheadr et al. [38]. However, these authors concluded that the strains may still be sensitive. Thus, strains with acquired resistance have not been detected until now. Using the recommended LSM-C medium [22] and the same [20] or similar conditions [19] for antimicrobial susceptibility testing, a chloramphenicol MIC ≤2 *μ*g/mL for one *B. thermophilum* strain [19] or MICs between 1 and 2 *μ*g/mL for ten *B. longum* strains [20] could be observed. As only a small number of strains were tested within the above-mentioned studies, the widening of the MIC distribution from 0.5 to 4 *μ*g/mL (Figure 1(a)) by investigating more than 100 strains in this study is obvious. Applying the recommended breakpoint of EFSA [11], all strains of the unimodal distribution are categorized as susceptible, approving the recent EFSA microbiological breakpoint for chloramphenicol.

Most bifidobacteria have been reported as resistant to aminoglycosides because of the lack of cytochrome-mediated drug transport system, resulting in a failure of the drug to reach its target [39]. The resistance of the tested strains to gentamicin and streptomycin has already been reported before [16, 17]. Correspondingly, all strains were resistant to kanamycin (128–>128 *μ*g/mL, Figure 1(b)) and neomycin (16–>128 *μ*g/mL, Figure 1(d)). The particular resistance of bifidobacteria to kanamycin is well known, and MICs between 64 and >1024 *μ*g/mL were described for this antimicrobial agent, whereas for neomycin MICs lay between 16 and >1024 *μ*g/mL testing a large number of species such as *B. bifidum*, *B. breve*, *B. catenulatum*, *B. infantis*, *B. longum*, *B. suis*, *B. thermophilum,* and others [32, 35–37]. Using the LSM-C medium, the antimicrobial susceptibility to kanamycin was only tested by Kushiro et al. [20] and Klose et al. [19]. Whereas Kushiro et al. [20] received MICs between 128 and 512 *μ*g/mL for ten *B. longum* strains, the tested *B. thermophilum* strain of Klose et al. [19] displayed an MIC value of 64 *μ*g/mL. The lower MIC value detected by Klose et al. may be due to other testing conditions (i.e., 24 hours instead of 48 hours of incubation), underlining the importance of controlled and standardized conditions for susceptibility testing [40]. While breakpoints for gentamicin and streptomycin are indicated in the EFSA document [11], none is required for kanamycin or specified for neomycin.

Low level, unimodal MIC distributions between 0.5 and 1 *μ*g/mL have been reported for linezolid and different bifidobacterial species, suggesting all bifidobacteria are susceptible to this antibiotic [37, 41]. This is in good accordance with our results, since all tested strains were inhibited by a linezolid concentration lower than 4 *μ*g/mL (Figure 1(c)). Ten *B. longum* strains, also tested using the same medium and conditions, displayed MIC values of 0.5 and 1 *μ*g/mL [20]. As the nonmutational resistance to linezolid, which is due to the acquisition of the *cfr* gene, is extremely rare and also confers resistance to chloramphenicol [42, 43], testing for this antimicrobial agent was no longer considered as relevant by EFSA [11]. Checking for chloramphenicol resistance should efficiently cover the hazard of an acquired resistance to linezolid [11].

The *in vitro* susceptibility of bifidobacteria to quinupristin-dalfopristin has been rarely studied. Only two previous reports showed that bifidobacteria are susceptible to this semisynthetic mixture by testing 100 strains of 11 bifidobacterial species by agar overlay disc diffusion [18] and one *B. thermophilum* strain by broth microdilution [19] using the same test medium (LSM-C). The obtained MIC value of 0.5 *μ*g/mL by Klose et al. [19] was one dilution step higher than the MIC range (≤0.12–0.25 *μ*g/mL) received within this study (Figure 1(e)). The EFSA breakpoint of 1 *μ*g/mL to quinupristin-dalfopristin appears to be applicable for bifidobacteria [11].

Reduced susceptibility of bifidobacteria towards trimethoprim was described by Ouoba et al. [37]. Susceptibility was also found to be variable and strain-specific by Masco et al. investigating 100 strains of 11 bifidobacterial species [18] or to be ≥32 *μ*g/mL by Kushiro et al. testing 10 *B. longum* strains [20], also using the same medium as within this study. Hence, a variable but species-specific susceptibility was detected, nearly covering the whole concentration range tested (Figure 1(f)). Also a wide range of trimethoprim MICs with no clear breakpoint values was identified for certain *Lactobacillus* species [44]. This was led back to the presence of antagonistic components in the medium, complicating susceptibility testing concerning trimethoprim [11, 15]. Thus, MIC testing of trimethoprim was no longer considered as relevant by EFSA [11].

## 4. Conclusion

In this study, the recently published ISO 10932/IDF 233 standard [22] was used to provide susceptibility data on the basis of a representative number of animal bifidobacteria. These data could be used for reviewing or verifying present microbiological breakpoints suggested by regulatory agencies to assess the safety of microorganisms intended for the use in probiotics. Nevertheless, more data including MIC values of human bifidobacteria for these antimicrobial agents applying the same testing conditions are needed to obtain adequate breakpoints for differentiating susceptible bacteria from those with acquired resistance. Additionally, a broad screening of resistance genes using molecular tools would also be of importance for the definition of applicable breakpoints. 

## Figures and Tables

**Table 1:** Susceptibility of 8 *B. animalis*, 36. *B. pseudolongum*, and 73 *B. thermophilum* strains to selected antimicrobial agents as determined by the broth microdilution method using LSM-C medium.

Antibiotic	Species	MIC (*μ*g/mL)		
		MIC range	MIC_50_	MIC_90_
Chloramphenicol	*B. animalis*	2–4	2	4
	*B. pseudolongum*	1-2	1	2
	*B. thermophilum*	0.5–2	1	2
Kanamycin	*B. animalis*	>128	>128	>128
	*B. pseudolongum*	≥128	>128	>128
	*B. thermophilum*	≥128	>128	>128
Linezolid	*B. animalis*	0.5–2	1	2
	*B. pseudolongum*	0.5–2	1	1
	*B. thermophilum*	0.5–1	0.5	1
Neomycin	*B. animalis*	32–>128	64	>128
	*B. pseudolongum*	16–>128	64	>128
	*B. thermophilum*	16–>128	128	>128
Quinupristin/	*B. animalis*	≤0.12	≤0.12	≤0.12
Dalfopristin	*B. pseudolongum*	≤0.12–0.25	≤0.12	0.25
	*B. thermophilum*	≤0.12–0.25	≤0.12	0.25
Trimethoprim	*B. animalis*	≤0.12	≤0.12	≤0.12
	*B. pseudolongum*	≤0.12–32	≤0.12	4
	*B. thermophilum*	0.5–>128	8	128

MIC_50_ and MIC_90_: MICs (*μ*g/mL) that inhibited 50% and 90% of the number of strains tested, respectively.
